# Sex-Specific Association between Metabolic Abnormalities and Elevated Alanine Aminotransferase Levels in a Military Cohort: The CHIEF Study

**DOI:** 10.3390/ijerph15030545

**Published:** 2018-03-19

**Authors:** Kai-Wen Chen, Fan-Chun Meng, Yu-Lueng Shih, Fang-Ying Su, Yen-Po Lin, Felicia Lin, Jia-Wei Lin, Wei-Kuo Chang, Chung-Jen Lee, Yi-Hwei Li, Chung-Bao Hsieh, Gen-Min Lin

**Affiliations:** 1 Department of Medicine, Hualien Armed Forces General Hospital, No. 100, Jin-Feng St., Hualien 970, Taiwan; az0127@gmail.com (K.-W.C.); flwoolol@gmail.com (F.L.); albert0920@yahoo.com.tw (C.-B.H.); 2 Departments of Medicine, Tri-Service General Hospital, National Defense Medical Center, Taipei 114, Taiwan; drking0724@gmail.com (F.-C.M.); albreb@ms28.hinet.net (Y.-L.S.); weikuohome@hotmail.com (W.-K.C.); 3 Department of Public Health, Tzu-Chi University, Hualien 970, Taiwan; 104324111@gms.tcu.edu.tw (F.-Y.S.); yihwei@mail.tcu.edu.tw (Y.-H.L.); 4 Department of Critical Care Medicine, Yonghe Cardinal Tien Hospital, Fu-Jen Catholic University, New Taipei City 234, Taiwan; b101093018@tmu.edu.tw; 5 Department of Dentistry, National Yang-Ming University, Taipei 112, Taiwan; ljw099533@gmail.com; 6 Department of Nursing, Tzu-Chi College of Technology, Hualien 970, Taiwan; guggilee@ems.tcust.edu.tw; 7 College of Science and Engineering, National Dong Hwa University, Hualien 974, Taiwan

**Keywords:** alanine aminotransferase, metabolic syndrome, military cohort, sex difference

## Abstract

The association of metabolic syndrome (MetS) components with elevated serum alanine aminotransferase (ALT) levels, a marker of hepatic injury, may differ between men and women. However, the sex-specific association in a military young population which has a low prevalence of MetS was unclear. We conducted a cross-sectional examination in 6738 men and 766 women, aged 18–50 years, from the cardiorespiratory fitness study in armed forces (CHIEF) in eastern Taiwan. The components of MetS were defined according to the updated International Diabetes Federation (IDF) ethnic criteria for Asians. Elevated ALT levels were defined as ≥40 U/L for both sexes and ≥30 U/L for women alternatively. Multivariate logistic regression analysis was performed to determine the sex-specific association between MetS components and elevated ALT. The prevalence of MetS and elevated ALT in men were 11.9% and 12.7% respectively, and in women were 3.5%, and 3.8% respectively. In men, high-density lipoprotein < 40 mg/dL, blood pressures ≥ 130/85 mmHg, serum triglycerides ≥ 150 mg/dL, and waist size ≥ 90 cm were associated with elevated ALT (odds ratios (OR) and 95% confidence intervals: 1.59 (1.34–1.90), 1.40 (1.19–1.65), 2.00 (1.68–2.39), and 1.68 (1.38–2.04); all *p* < 0.001); whereas in women, only fasting plasma glucose ≥ 100 mg/dL was associated with elevated ALT ≥ 40 U/L (OR: 7.59 (2.35–24.51), *p* = 0.001) and ALT ≥ 30 U/L (2.67 (0.89–7.95), *p* = 0.08). Our findings suggest that the relationship between metabolic abnormalities and elevated ALT may differ by sex, possibly due to the MetS more prevalent in young adult men than in women.

## 1. Introduction 

Non-alcoholic fatty liver disease (NAFLD), characterized by excessive accumulation of hepatic fat, is defined as the presence of steatosis in more than 5% of hepatocytes [1]. NAFLD affects 17–60% of adults in different countries and the prevalence in Taiwan is estimated to be 11.5% to 57.8% [2,3]. Today, NAFLD has become a leading etiology of chronic liver disease worldwide [4]. Nonalcoholic steatohepatitis (NASH) is a more aggressive form of NAFLD and covers a wide spectrum of disease severity, including hepatitis, fibrosis, cirrhosis, and hepatocellular carcinoma [5]. NASH commonly coexists with elevated markers of liver injury, particularly serum alanine aminotransferase (ALT), which is closely related to the severity of fat accumulation in the liver and frequently used as a marker of NAFLD in relation to type 2 diabetes mellitus in several epidemiology studies [6,7]. 

Metabolic syndrome (MetS) is defined as central obesity occurring in parallel with two of the following components: (1) raised levels of blood pressures, triglycerides, and fasting plasma glucose; and (2) reduced high-density lipoprotein concentration based on the International Diabetes Federation (IDF) criteria [8]. In addition, obesity, diabetes mellitus, insulin resistance, dyslipidemia, and hypertension may lead to NAFLD, which has been considered as the liver’s manifestation of MetS [9]. Prospective studies have also shown that elevated ALT levels related to NAFLD or NASH is associated with the occurrence of new-onset MetS and type 2 diabetes, independent of age, obesity, and alcohol intake [10,11]. 

Previous studies reported sex differences in the prevalence of NAFLD, with it affecting more men than women [12]. This finding was also reflected in the distribution of visceral fat, where women have less visceral fat than men [13]. Similarly, the prevalence and components of MetS may also differ by sex [14]. Studies had suggested that female hormones could protect against visceral fat accumulation and metabolic abnormalities, leading to a lower prevalence of MetS and hepatic injury related to NAFLD among premenopausal women [15]. Since there were few studies investigating the association of MetS components and elevated ALT levels between young men and women, we decided to examine the sex-specific association in a large military cohort in Taiwan.

## 2. Materials and Methods 

### 2.1. Study Population

From January 2013 to December 2014, there were 9076 military participants enrolled in the cardiorespiratory fitness and hospitalization events in armed forces (CHIEF) study. We further excluded participants who had unavailable relevant covariates and those with viral hepatitis and without a ultrasound-proven fatty infiltration of liver, leaving a final sample of 7504 individuals (82.7%) including 6738 men and 766 women, ages between 18 and 50 years for analysis. The study design has been described in detail previously [16]. In brief, each participant was asked to self-report a questionnaire including demographic information, medical history, current cigarette smoking habits, and alcohol consumption. All participants underwent physical examinations, anthropometric measurements for height, weight, and waist circumference at standing position, and hemodynamic status of pulse rate and blood pressures, which were automatically measured by the PARAMA TECH FT-201 blood pressure monitor over the right upper arm at sitting position, after taking a rest for at least 15 min. Blood tests were performed for concentrations of fasting plasma glucose, triglycerides, total cholesterol, high-density lipoprotein, low-density lipoprotein, serum uric acid, aspartate transaminase (AST), and alanine transaminase (ALT) in the Hualien Armed Forces General Hospital which is the only military referral center in Hualien, Taiwan, able to perform the whole body health exams. 

### 2.2. Definitions

In our analyses, elevated ALT levels were defined as ≥40 U/L for both sexes and ≥30 U/L for women alternatively [17]. The updated IDF definition of MetS includes the major component of waist size ≥90 cm for Asian men and ≥80 cm for Asian women in addition to two or more of the four minor components: (1) serum triglycerides ≥ 150 mg/dL; (2) high-density lipoprotein <40 mg/dL for men and <50 mg/dL for women; (3) systolic blood pressure ≥ 130 mmHg, or diastolic blood pressure ≥ 85 mmHg, or use of antihypertensive medication; (4) fasting plasma glucose ≥ 100 mg/dL, or use of antidiabetic medication [9]. Alternatively, the National Cholesterol Education Program Adult Treatment Panel (*NCEP ATP III*) guideline for MetS for men and women was defined as the presence of three or more of the previously mentioned major and minor criteria [18]. Body mass index was defined as weight (kg)/square of height (m^2^). Obese, overweight, normal-weight, and underweight were defined by a body mass index ≥30, 25–29.9, 18–24.9, and <18 kg/m^2^, respectively. This study was reviewed and approved by the Institutional Review Board of the Mennonite Christian Hospital in Taiwan and written informed consent was obtained from all participants.

### 2.3. Statistical Analysis

Demographic characteristics and laboratory data are presented as means ± standard deviations or percent for continuous and categorical variables, respectively. Chi-square or Fisher’s exact tests for categorical variables and Student’s *t*-tests for continuous variables were used between the two groups (normal levels of ALT vs. elevated ALT, and women vs. men). Univariate logistic regression was used to identify the risk factors for elevated ALT based on sex. Multivariate logistic regression analysis was also performed in men and women for those variables with statistically significance in the univariate analysis to determine the association between the MetS components and elevated ALT. A two-tailed value of *p* < 0.05 was considered statistically significant. Analyses were performed using SAS statistical software (version 9.4, SAS Institute Inc., Cary, NC, USA). 

### 2.4. Ethical Approval

All procedures performed in studies involving human participants were in accordance with the ethical standards of the institutional and/or national research committee and with the 1964 Helsinki declaration and its later amendments or comparable ethical standards (Mennonite Christian Hospital Institutional Review Board/Ethics Committee approval code: 16-05-008). 

## 3. Results

### 3.1. Descriptive Characteristics

The baseline characteristics of those with and without elevated ALT ≥ 40 U/L are shown in Table 1. The mean age of overall participants was 29 years. Participants with elevated ALT were relatively older, more likely to be male, and had more prevalent obesity, current cigarette smoking, current alcohol intake, metabolic abnormalities, and an ALT/AST ratio > 1, an indicator of hepatic injury not caused by alcohol toxicity. The prevalence of IDF- and NCEP-ATP III-defined MetS was estimated to be 11.1% and 13.6% respectively in the overall cohort and much higher in those with elevated ALT than those without. Table S1 shows similar results if elevated ALT was defined as ≥30 U/L for women alternatively.

### 3.2. Sex Differences

**Figure 1:**
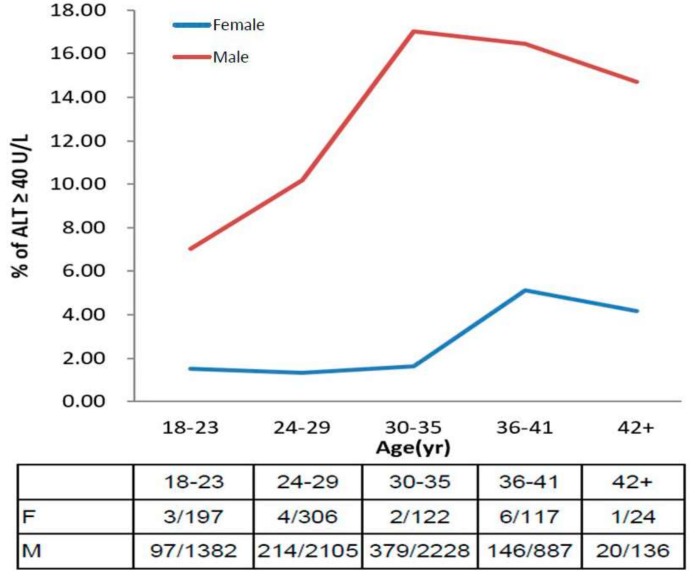
The age-based prevalence of elevated ALT in men and women.

The prevalence of elevated ALT ≥ 40 U/L in men and women stratified by age are shown in Figure 1. In general, elevated ALT levels were much more prevalent in men than women (12.7% vs. 2.1%). The prevalence of elevated ALT in men at ages of 18–23 years was 7.0% and increased sharply to the peak of 17% at ages of 30–35 years. By contrast, the prevalence in women at ages of 18–35 years was estimated only 1.3–1.6% and rose to the peak of 5.1% at ages of 36–41 years. Figure S1 shows that the prevalence of elevated ALT ≥ 30 U/L in women was estimated 3.8% and the peak was 9.4% at ages of 36–41 years.

The baseline characteristics of men and women are shown in Table 2. Men were modestly older and had more prevalent obesity, current smoking, current alcohol consumption, metabolic abnormalities, elevated ALT, and ALT/AST ratio > 1 compared with women. The prevalence of IDF- and NCEP-ATP III-defined MetS in men is 11.9% and 14.7% respectively, which are much higher than the 3.5% and 4.1% found in women. 

Table 3 shows the results of univariate analysis for men and women. In men, all variables including older age (odds ratio (OR) per five-year increase: 1.30), metabolic risk factors, and current alcohol intake (OR: 1.20) were associated with elevated ALT ≥ 40 U/L. The strongest associations in the metabolic components were waist circumference ≥90 cm or obesity (OR: 4.94 and 5.27 respectively) and MetS (OR: 5.36 and 5.81 according to the NCEP-ATP III and IDF based-criteria respectively). In contrast, for women, two of the metabolic risk factors—elevated blood pressures and total cholesterol, as well as current alcohol intake—were not associated with elevated ALT. The strongest associations in the metabolic components were fasting plasma glucose ≥100 mg/dL (OR: 12.6) and MetS (OR: 23.5 and 20.8 based on the NCEP-ATP III and IDF based-criteria respectively). Table S2 shows consistent results for women if elevated ALT was defined as ≥30 U/L, alternatively. The strongest associations in the metabolic components were fasting plasma glucose ≥100 mg/dL (OR: 5.39, 95% confidence intervals (CI): 2.06–14.12), serum triglycerides ≥150 mg/dL (OR: 5.54, 95% CI: 2.24–13.75), and MetS (OR: 14.63, 95% CI: 5.98–35.75 and 14.40, 95% CI: 5.66–36.60 based on the NCEP-ATP III and IDF based criteria, respectively).

Table 4 presents the multivariate logistic regression analyses results using age, body mass index category, total cholesterol concentrations, and the five MetS components to predict elevated ALT ≥ 40 U/L for the overall, male, and female groups, respectively. As expected in the overall group, men were more likely to have elevated ALT than women (OR: 3.86). In men, the strongest associations in the metabolic risk factors were being overweight and obesity (OR: 2.58 and 4.54 respectively) and serum triglycerides ≥ 150 mg/dL (OR: 2.00). By contrast in women, although obesity and waist circumference ≥ 80 cm were associated with two-fold higher risk of elevated ALT (OR: 2.24 and 2.37 respectively), the levels of statistical significance were not attained. Only fasting plasma glucose ≥ 100 mg/dL (OR: 7.59) was associated with elevated ALT levels ≥ 40 U/L in women. Table S3 shows that both fasting plasma glucose ≥ 100 mg/dL and serum triglycerides ≥ 150 mg/dL were borderline associated with elevated ALT levels ≥ 30 U/L in women (OR: 2.67, 95% CI: 0.89–7.95 and 2.77, 95% CI: 0.95–8.12, respectively). 

## 4. Discussion

This is the first cross-sectional cohort study focusing on the prevalence of MetS and the metabolic risk factors for abnormal levels of liver enzyme in young military men and women in Taiwan. We found a low prevalence of MetS in men and women which is consistent with that in other young Asian adults (14–15.6% in men and 2.3–5.4% in women) [19,20]. Furthermore, we identified that the association between MetS components and elevated ALT levels might differ by sex in young adults. 

Metabolic abnormalities associated with hepatic injury and elevated ALT levels have been reported in previous studies for other Asian populations [19,20,21]. In the present study, an ALT/AST ratio >1 was found in more than 97% of those with elevated ALT, presumably from NAFLD [22]. We also noticed that young adults appeared to be at risk of having elevated ALT levels, especially in the age 30–40 group. Schmucker [23] reported that these age-related liver function changes can be explained by change with age in liver volume, hepatic dense body compartment, shifts in the expression of a variety of proteins, a lower inflammatory response to oxidative stress, diminished hepatobiliary functions, and increased fibrosis. In addition, Dong et al. [24] demonstrated that ALT levels decreased with age, independent of sex, alcohol use, and metabolic risk factors in a longitudinal follow-up. 

The association of the MetS components with elevated ALT may differ by sex. Previous studies have shown that prevalence of NAFLD in non-diabetic men was higher than that in non-diabetic women in an Asian population [25], and this was consistent with the finding in another study of a U.S. adult cohort [26]. These data suggested that diabetes or high fasting plasma glucose in women is highly correlated with NAFLD and inflammation status on liver. Liver-fat accumulation or NAFLD was also reported with an association with insulin resistance in women [27,28]. Moreover, Levitzky et al. uncovered that impaired fasting glucose levels in women were associated with higher risk of coronary heart disease risk, which may share a similar pathogenesis with NAFLD [29], whereas this was not shown in men. By contrast, the association of obesity dyslipidemia, and hypertension rather than abnormal fasting plasma glucose with elevated ALT seemed to be stronger in men. The mechanism for the difference in association based on sex is not clear and needs further investigation. 

Due to our female population being mostly at premenopausal ages, the prevalence of MetS and elevated ALT were particularly low. This result was also shown in other epidemiology studies [19,30]. Hamaguchi et al. have revealed an association of the postmenopausal state with NAFLD and indicated that the postmenopausal state was a risk factor for NAFLD [31]. Estrogen can provide many benefits in body metabolism such as maintaining proper fluid balance, increasing high density lipoproteins and decreasing low density lipoproteins. Female estrogens also have many benefits for the liver such as inhibition of fibrogenesis, promotion of antioxidant effects, increase in innate immunity, inhibition of cellular senescence, and protection of mitochondrial structure and function [32]. Loss of estrogen has been associated with an increase of central fat [33]. In addition, Grobe et al. found women without NAFLD had higher levels of serum estradiol compared with NAFLD patients [15]. Therefore female hormones, especially estrogen, may have a protective effect of against NAFLD in women. 

This study has several limitations. First, behavioral characteristics such as smoking, drinking, and physical activity are collected by self-report and lack of quantitative measures, which may result in some errors and confounding effects. Second, the temporal association between the five MetS components and elevated ALT levels could not be made as a cross-sectional design in nature. Third, there were only 766 women compared to 6738 men in the present study. With this relatively limited sample size of the female subjects, it is important to note that the conclusions drawn from women were statistically less powerful and evident, and deserve further study to verify our findings. 

## 5. Conclusions

The relationship between metabolic abnormalities with elevated ALT levels may differ by sex in young military adults below 50 years of age in Taiwan. Fasting plasma glucose ≥ 100 mg/dL was more specific to elevated ALT in women, whereas obesity, dyslipidemia, and hypertension were more specific to elevated ALT in men. The mechanisms for the sex difference might be related to the MetS and current alcoholic intake more prevalent in young adult men than in women.

## Figures and Tables

**Table 1:** Baseline characteristics of the study cohort based on serum ALT levels.

	Overall	ALT < 40	ALT ≥ 40 U/L	*p*-Value
	N = 7504	N = 6632	N = 872	
Age (year)	28.93 ± 6.04	28.69 ± 6.06	30.74 ± 28.35	<0.0001
Specialty, %				<0.0001
Air forces	19.75	1.66	0.34	
Army	78.74	79.52	72.82	
Navy	1.51	18.82	26.83	
SEX, %				<0.0001
Women	10.21	11.31	1.83	
Men	89.79	88.69	98.17	
BMI (kg/m^2^), %	24.96 ± 3.72	24.52 ± 3.48	28.35 ± 3.77	<0.0001
Underweight (<18.5)	2.00	2.22	0.34	<0.0001
Normal (18.5–24.9)	51.88	56.35	17.89	
Overweight (25–29.9)	37.34	35.13	54.13	
Obesity (≥30)	8.78	6.30	27.64	
Current smoker, %	33.81	32.78	41.63	<0.0001
Current alcohol intake, %	45.19	44.38	51.38	<0.0001
ALT/AST ratio ≥ 1, %	47.23	40.65	97.25	<0.0001
Elevated blood pressure, % *	25.61	23.21	43.92	<0.0001
Waist circumference, % ^∫^	27.60	23.33	60.09	<0.0001
Serum TG ≥ 150 mg/dL, %	19.95	16.34	47.36	<0.0001
FPG ≥ 100 mg/dL, %	14.43	13.31	22.94	<0.0001
Low serum HDL, % ^$^	21.16	18.97	37.84	<0.0001
Total cholesterol ≥ 200 mg/dL, %	20.26	17.84	38.65	<0.0001
Metabolic syndrome (ATPIII), %	13.58	10.12	39.91	0.0001
Metabolic syndrome (IDF), %	11.06	7.89	35.21	<0.0001

Continuous variables are expressed as mean ± standard deviation and categorical variables as number (percentage). Abbreviations: ALT, alanine aminotransferase; AST, aspartate aminotransferase; ATP III, National Cholesterol Education Program Adult Treatment Panel III; BMI, body mass index; FPG, fasting plasma glucose; HDL, high density lipoprotein; IDF, International Diabetes Federation; TG, triglycerides. * Elevated blood pressure: blood pressure ≥ 130/85 mm Hg or use of antihypertensive agents. ^∫^ Waist circumference: ≥90 cm in men and ≥80 cm in women. ^$^ Low serum HDL: <40 mg/dL in men and <50 mg/dL in women.

**Table 2:** Baseline characteristics of the study cohort of men and women.

Variables	Women	Men	*p*-Value
	N = 766	N = 6738	
Age (year)	28.03 ± 6.61	29.03 ± 5.97	<0.0001
Specialty, %			<0.0001
Air forces	1.04	1.56	
Army	85.51	77.98	
Navy	13.45	20.47	
BMI (kg/m^2^), %	22.59 ± 3.11	25.23 ± 3.69	<0.0001
Underweight (<18.5)	6.01	1.54	<0.0001
Normal (18.5–24.9)	72.98	49.48	
Overweight (25–29.9)	18.80	39.45	
Obesity (≥30)	2.22	9.53	
Current smoker, %	10.18	36.49	<0.0001
Current alcohol intake, %	21.80	47.85	<0.0001
ALT, %			<0.0001
<40 U/L	97.91	87.30	
≥40 U/L	2.09	12.70	
ALT/AST ratio ≥ 1, %	14.75	50.92	<0.0001
Elevated blood pressure, % *	6.01	27.84	<0.0001
Waist circumference, % ^∫^	23.37	28.08	0.0057
Serum TG ≥ 150 mg/dL, %	6.14	21.52	<0.0001
FPG ≥ 100 mg/dL, %	5.22	15.48	<0.0001
Low serum HDL, % ^$^	25.98	20.61	0.0006
Total cholesterol ≥ 200 mg/dL, %	14.36	20.93	<0.0001
Metabolic syndrome (ATPIII), %	4.05	14.66	<0.0001
Metabolic syndrome (IDF), %	3.52	11.92	<0.0001

Continuous variables are expressed as mean ± standard deviation and categorical variables as number (percentage). Abbreviations: ALT, alanine aminotransferase; AST, aspartate aminotransferase; ATP III, National Cholesterol Education Program Adult Treatment Panel III; BMI, body mass index; FPG, fasting plasma glucose; HDL, high density lipoprotein; IDF, International Diabetes Federation; TG, triglycerides. * Elevated blood pressure: blood pressure ≥ 130/85 mm Hg or use of antihypertensive agents. **^∫^** Waist circumference: ≥90 cm in men and ≥80 cm in women. **^$^** Low serum HDL: <40 mg/dL in men and <50 mg/dL in women.

**Table 3:** Univariate analysis of risk factors predicting elevated ALT ≥ 40 U/L based on sex.

Variables	Overall	*p*-Value	Women	*p*-Value	Men	*p*-Value
	N = 7504		N = 766		N = 6738	
	OR (95% CI)		OR (95% CI)		OR (95% CI)	
Age (by five-year increment)	1.31 (1.24–1.39)	<0.0001	1.36 (0.97–1.89)	0.074	1.30 (1.23–1.38)	<0.0001
BMI (kg/m^2^)						
25–29.9	2.18 (1.89–2.51)	<0.0001	2.66 (0.95–7.45)	0.062	2.01 (1.74–2.32)	<0.0001
≥30	5.68 (4.75–6.79)	<0.0001	7.00 (1.46–33.6)	0.015	5.27 (4.40–6.31)	<0.0001
Elevated blood pressure *	2.59 (2.24–3.00)	<0.0001	2.29 (0.51–10.4)	0.28	2.36 (2.03–2.73)	<0.0001
Waist circumference ^∫^	4.95 (4.27–5.73)	<0.0001	4.39 (1.61–12.0)	0.0038	4.94 (4.26–5.74)	<0.0001
Serum TG ≥ 150 mg/dL	4.61 (3.97–5.34)	<0.0001	3.70 (1.02–13.5)	0.047	4.28 (3.68–4.97)	<0.0001
FPG ≥ 100 mg/dL	1.94 (1.63–2.30)	<0.0001	12.64 (4.34–36.8)	<0.0001	1.74 (1.46–2.07)	<0.0001
Low serum HDL ^$^	2.60 (2.24–3.02)	<0.0001	2.93 (1.08–7.91)	0.034	2.72 (2.33–3.17)	<0.0001
Total cholesterol ≥ 200 mg/dL	2.90 (2.50–3.37)	<0.0001	1.39 (0.39–4.95)	0.61	2.86 (2.45–3.33)	<0.0001
Current alcohol intake	1.32 (1.15–1.53)	<0.0001	1.20 (0.38–3.77)	0.75	1.20 (1.04–1.39)	0.012
Metabolic syndrome (ATPIII)	5.90 (5.04–6.91)	<0.0001	23.53 (8.09–68.5)	<0.0001	5.36 (4.57–6.28)	<0.0001
Metabolic syndrome (IDF)	6.35 (5.38–7.49)	<0.0001	20.83 (6.93–62.64)	<0.0001	5.81 (4.92–6.87)	<0.0001

Data are expressed as odds ratio (OR) and 95% confidence intervals (CI). Abbreviations: ATP III, National Cholesterol Education Program Adult Treatment Panel III; BMI, body mass index; FPG, fasting plasma glucose; HDL, high density lipoprotein; IDF, International Diabetes Federation; TG, triglycerides. * Elevated blood pressure: blood pressure ≥ 130/85 mm Hg or use of antihypertensive agents. **^∫^** Waist circumference: ≥90 cm in men and ≥80 cm in women. **^$^** Low serum HDL: <40 mg/dL in men and <50 mg/dL in women.

**Table 4:** Multivariate analysis of the metabolic risk factors predicting elevated ALT ≥ 40 U/L based on sex.

Variables	Overall	*p*-Value	Women	*p*-Value	Men	*p*-Value
	N = 7504		N = 766		N = 6738	
	OR (95% CI)		OR (95% CI)		OR (95% CI)	
Sex (men vs. women)	3.86 (2.31–6.46)	<0.0001	N/A		N/A	
Age (by five-year increment)	1.03 (0.96–1.10)	0.46	1.16 (0.79–1.72)	0.46	1.02 (0.95–1.10)	0.53
BMI (kg/m^2^)						
25–29.9	2.53 (2.03–3.15)	<0.0001	1.24 (0.30–5.17)	0.77	2.58 (2.06–3.22)	<0.0001
≥30	4.46 (3.32–5.99)	<0.0001	2.24 (0.27–18.84)	0.46	4.54 (3.37–6.13)	<0.0001
Serum TG ≥ 150 mg/dL	1.99 (1.67–2.37)	<0.0001	1.31 (0.30–5.79)	0.73	2.00 (1.68–2.39)	<0.0001
FPG ≥ 100 mg/dL	1.10 (0.90–1.33)	0.35	7.59 (2.35–24.51)	0.001	1.05 (0.86–1.28)	0.62
Elevated blood pressure *	1.40 (1.19–1.64)	<0.0001	1.15 (0.20–6.74)	0.87	1.40 (1.19–1.65)	<0.0001
Waist circumference ^∫^	1.69 (1.39–2.05)	<0.0001	2.37 (0.62–9.15)	0.21	1.68 (1.38–2.04)	<0.0001
Low serum HDL ^$^	1.61 (1.35–1.92)	<0.0001	1.71 (0.52–5.60)	0.37	1.59 (1.34–1.90)	<0.0001
Total cholesterol ≥ 200 mg/dL	1.81 (1.53–2.16)	<0.0001	0.95 (0.21–4.20)	0.94	1.84 (1.55–2.19)	<0.0001

Data are expressed as odds ratio (OR) and 95% confidence intervals (CI). Abbreviations: BMI, body mass index; FPG, fasting plasma glucose; HDL, high density lipoprotein; N/A, not available; TG, triglycerides. * Elevated blood pressure: blood pressure ≥ 130/85 mm Hg or use of antihypertensive agents. **^∫^** Waist circumference: ≥90 cm in men and ≥80 cm in women. **^$^** Low serum HDL: <40 mg/dL in men and <50 mg/dL in women.

## Supplementary Materials

The following are available online at http://www.mdpi.com/1660-4601/15/3/545/s1, Table S1: Baseline Characteristics of the Study Cohort based on Serum ALT levels, Table S2: Univariate Analysis of Risk Factors Predicting Elevated ALT ≥30U/L in Women, Table S3: Multivariate Analysis of the Risk Factors Predicting Elevated ALT ≥30 U/L based on Sex, Figure S1: The age-based prevalence of elevated ALT in men and women. ^#^Elevated ALTs are defined as ALT level ≥ 40U/L in men and ≥30 U/L in women.
