# Supplementary material for: Sex-Specific Association between Metabolic Abnormalities and Elevated Alanine Aminotransferase Levels in a Military Cohort: The CHIEF Study
Source: Int J Environ Res Public Health. 2018 Mar 19;15(3):545. doi: 10.3390/ijerph15030545 (PMC5877090; doi:10.3390/ijerph15030545)
Supplement: Supplementary file 1 [file ijerph-15-00545-s001.pdf]

Supplementary Materials: Sex-Specific Association between Metabolic Abnormalities and Elevated Alanine Aminotransferase Levels in a Military Cohort: The CHIEF Study

Kai-Wen Chen, Fan-Chun Meng, Yu-Lueng Shih, Fang-Ying Su, Yen-Po Lin, Felicia Lin, Jia-Wei Lin, Wei-Kuo Chang, Chung-Jen Lee, Yi-Hwei Li, Chung-Bao Hsieh and Gen-Min Lin *

Table S1. Baseline Characteristics of the Study Cohort based on Serum ALT levels.

Variables	Overall N = 7504	Normal ALT N = 6619	Elevated ALT N = 885	*p*-value
Age (year)	28.93 ± 6.04	28.69 ± 6.06	30.75 ± 5.65	<0.0001
Specialty, %				<0.0001
Air forces	19.75	1.66	0.34	
Army	78.74	79.52	72.88	
Navy	1.51	18.82	26.78	
SEX, %				<0.0001
Women	10.21	11.13	3.28	
Men	89.79	88.87	96.72	
BMI (kg/m^2), %	24.96 ± 3.72	24.51 ± 3.47	28.33 ± 3.78	<0.0001
Underweight (<18.5)	2.00	2.22	0.34	<0.0001
Normal (18.5-24.9)	51.88	56.38	18.19	
Overweight (25–29.9)	37.34	35.11	54.01	
Obesity (≥30)	8.78	6.28	27.46	
Current smoker, %	33.81	32.78	41.63	<0.0001
Current alcohol intake, %	45.19	44.45	50.73	<0.0001
ALT/AST ratio ≥ 1, %	47.23	40.53	97.29	<0.0001
Elevated blood pressure, % *	25.61	23.19	43.73	<0.0001
Waist circumference, % ʃ	27.60	23.25	60.11	<0.0001
Serum TG ≥150 mg/dL, %	19.95	16.32	47.12	<0.0001
FPG ≥100 mg/dL, %	14.43	13.34	22.60	<0.0001
Low serum HDL, % $	21.16	18.92	37.97	<0.0001
Total cholesterol ≥200 mg/dL, %	20.26	17.83	38.42	<0.0001
Metabolic syndrome (ATPIII), %	13.58	10.11	39.55	0.0001
Metabolic syndrome (IDF), %	11.06	7.87	34.92	<0.0001

Elevated ALTs are defined as alanine aminotransferase (ALT) level ≥ 40U/L in men and ≥30 U/L in women. Continuous variables are expressed as mean ± standard deviation and categorical variables as number (percentage). Abbreviations: ALT, alanine aminotransferase; AST, aspartate aminotransferase; ATP III, National Cholesterol Education Program Adult Treatment Panel III; BMI, body mass index; FPG, fasting plasma glucose; HDL, high density lipoprotein; IDF, International Diabetes Federation; TG, triglycerides. * Elevated blood pressure: blood pressure ≥130/85 mm Hg or use of antihypertensive agents. ʃ Waist circumference: ≥90 cm in men and ≥80 cm in women. $ Low serum HDL: <40 mg/dL in men and <50 mg/dL in women.

Table S2. Univariate Analysis of Risk Factors Predicting Elevated ALT ≥ 30 U/L in Women.

Variables	Women N = 766 OR (95% CI)	*p*-value
Age (by 5 yr increment)	1.42 (1.11–1.82)	0.006
BMI (kg/m^2)		
25–29.9	4.14 (1.85–9.28)	<0.0001
≥30	14.01 (4.02–48.82)	<0.0001
Serum TG ≥150 mg/dL	5.54 (2.24–13.75)	<0.0001
FPG ≥100 mg/dL	5.39 (2.06–14.12)	0.001
Elevated blood pressure *	4.55 (1.75–11.79)	0.002
Waist circumference ʃ	5.03 (2.35–10.74)	<0.0001
Low serum HDL $	2.79 (1.32–5.88)	0.007
Total cholesterol ≥200 mg/dL	1.59 (0.63–3.99)	0.33
Current alcohol intake	0.74 (0.28–1.97)	0.55
Metabolic syndrome (ATPIII)	14.63 (5.98–35.75)	<0.0001
Metabolic syndrome (IDF)	14.40 (5.66–36.60)	<0.0001

Data are expressed as odds ratio (OR) and 95% confidence intervals (CI). Abbreviations: ATP III, National Cholesterol Education Program Adult Treatment Panel III; BMI, body mass index; FPG, fasting plasma glucose; HDL, high density lipoprotein; IDF, International Diabetes Federation; TG, triglycerides.
* Elevated blood pressure: blood pressure ≥ 130/85 mm Hg or use of antihypertensive agents. ʃ Waist circumference: ≥90 cm in men and ≥80 cm in women. $ Low serum HDL: <40 mg/dL in men and <50 mg/dL in women.

Table S3. Multivariate Analysis of the Risk Factors Predicting Elevated ALT ≥ 30 U/L based on Sex.

	Overall N = 7504 OR (95% CI)	*p*-value	Women N = 766 OR (95% CI)	*p*-value	Men N = 6738 OR (95% CI)	*p*-value
Sex (men vs women)	2.03 (1.37–3.02)	<0.0001	N/A		N/A	
Age (by 5 yr increment)	1.03 (0.96–1.10)	0.36	1.16 (0.86–1.57)	0.32	1.02 (0.95–1.10)	0.53
BMI (kg/m^2)						
25–29.9	2.53 (2.04–3.15)	<0.0001	1.48 (0.50–4.34)	0.47	2.58 (2.06–3.22)	<0.0001
≥30	4.48 (3.34–6.00)	<0.0001	3.53 (0.71–17.52)	0.12	4.54 (3.37–6.13)	<0.0001
Serum TG ≥150 mg/dL	2.00 (1.68–2.38)	<0.0001	2.77 (0.95–8.12)	0.06	2.0 (1.68–2.39)	<0.0001
FPG ≥100 mg/dL	1.08 (0.90–1.33)	0.45	2.67 (0.89–7.95)	0.08	1.05 (0.86–1.28)	0.62
Elevated blood pressure *	1.40 (1.20–1.66)	<0.0001	2.47 (0.78–7.80)	0.12	1.40 (1.19–1.65)	<0.0001
Waist circumference ʃ	1.69 (1.39–2.05)	<0.0001	2.34 (0.84–6.50)	0.10	1.68 (1.38–2.04)	<0.0001
Low serum HDL $	1.62 (1.36–1.92)	<0.0001	1.69 (0.69–4.12)	0.25	1.59 (1.34–1.90)	<0.0001
Total cholesterol ≥200 mg/dL	1.80 (1.52–2.13)	<0.0001	0.85 (0.28–2.60)	0.78	1.84(1.55–2.19)	<0.0001

Data are expressed as odds ratio (OR) and 95% confidence intervals (CI). Abbreviations: BMI, body mass index; FPG, fasting plasma glucose; HDL, high density lipoprotein; IDF, International Diabetes Federation; TG, triglycerides. * Elevated blood pressure: blood pressure ≥130/85 mm Hg or use of antihypertensive agents. ʃ Waist circumference: ≥90 cm in men and ≥80 cm in women. $ Low serum HDL: <40 mg/dL in men and <50 mg/dL in women.

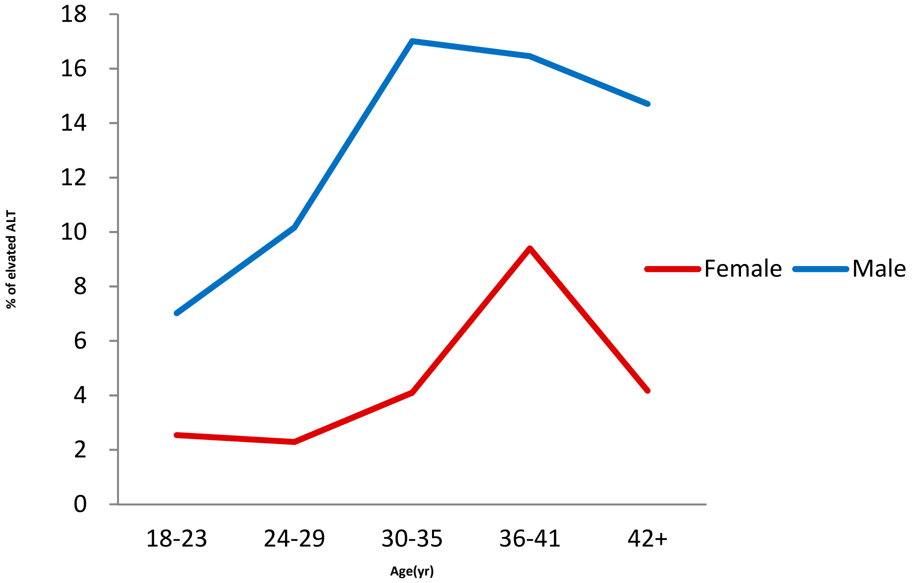

	18-23	24-29	30-35	36-41	42+
F	5/197	7/306	5/122	11/117	1/24
M	97/1382	214/2105	379/2228	146/887	20/136

Figure S1. The age-based prevalence of elevated ALT in men and women. [#]Elevated ALTs are defined as ALT level ≥ 40U/L in men and ≥30 U/L in women.
